# A Time-Distributed Spatiotemporal Feature Learning Method for Machine Health Monitoring with Multi-Sensor Time Series

**DOI:** 10.3390/s18092932

**Published:** 2018-09-03

**Authors:** Huihui Qiao, Taiyong Wang, Peng Wang, Shibin Qiao, Lan Zhang

**Affiliations:** 1 Key Laboratory of Mechanism Theory and Equipment Design of Ministry of Education, Tianjin University, Tianjin 300350, China; huihuiqiao@tju.edu.cn (H.Q.); pengwang@tju.edu.cn (P.W.); tju_zhanglan@tju.edu.cn (L.Z.); 2 School of Mechanical Engineering, Tianjin University, Tianjin 300354, China; 3 Institute for Special Steels, Central Iron and Steel Research Institute, Beijing 100081, China; zjjhxmd29@tju.edu.cn

**Keywords:** multi-sensor time series, deep learning, machine health monitoring, time-distributed ConvLSTM model, spatiotemporal feature learning

## Abstract

Data-driven methods with multi-sensor time series data are the most promising approaches for monitoring machine health. Extracting fault-sensitive features from multi-sensor time series is a daunting task for both traditional data-driven methods and current deep learning models. A novel hybrid end-to-end deep learning framework named Time-distributed ConvLSTM model (TDConvLSTM) is proposed in the paper for machine health monitoring, which works directly on raw multi-sensor time series. In TDConvLSTM, the normalized multi-sensor data is first segmented into a collection of subsequences by a sliding window along the temporal dimension. Time-distributed local feature extractors are simultaneously applied to each subsequence to extract local spatiotemporal features. Then a holistic ConvLSTM layer is designed to extract holistic spatiotemporal features between subsequences. At last, a fully-connected layer and a supervised learning layer are stacked on the top of the model to obtain the target. TDConvLSTM can extract spatiotemporal features on different time scales without any handcrafted feature engineering. The proposed model can achieve better performance in both time series classification tasks and regression prediction tasks than some state-of-the-art models, which has been verified in the gearbox fault diagnosis experiment and the tool wear prediction experiment.

## 1. Introduction

Accurate and real-time monitoring of machine health status has great significance. Appropriate maintenance strategies can be adopted depending on the real-time health status of the machine to avoid catastrophic failures, shorten downtime and reduce economic losses. Machine health monitoring (MHM) is of great significance to ensure the safety and reliability of equipment operation. Modern complex machinery systems, such as CNC machining equipment and trains, are moving in the direction of large-scale, complex, high-precision, reliable and intelligent. Moreover, the features of the signal to be processed vary with different devices, different operating conditions and different fault conditions [1]. Therefore, it puts forward higher requirements for the accuracy, efficiency and versatility of condition monitoring and fault diagnosis methods.

With the rapid development of advanced sensing technology and affordable storage, it is much easier to acquire mechanical condition data, enabling large scale collection of time series data. With the massive monitoring data, data-driven methods have been greatly developed and applied in the field of MHM. The framework of the traditional data-driven MHM system includes four steps: signal acquisition, feature extraction, feature reduction and condition classification or prediction [2,3]. 

The types of sensor signal commonly used in MHM systems include: vibration signal, acoustic emission signal, force, rotational speed, current signal, power signal, temperature and so forth. Different types of sensors and measuring positions are sensitive to different types of damage and have different advantages and limitations. Multi-sensor fusion data that contains redundant and complementary mechanical health information can help the MHM system to achieve higher diagnostic accuracy and detect more failures than a single type of sensor data [4]. The monitoring values of each sensor channel constitute a 1D temporal sequence in chronological order. If the outputs of a plurality of sensor channels are arranged in parallel and all channels have the same sampling rate, a 2D spatiotemporal sequence data is formed. Since multiple measuring channels with high sampling frequency and long data collection period are required for each mechanical functional component, the 2D spatiotemporal sequence not only includes the local spatial-domain dependency between different channels but also includes the time-domain dependency of each channel data, which presents complex temporal correlation and spatial correlation. In addition, the 2D spatiotemporal sequence is dynamic, non-linear, multivariable, high redundant and strong noisy, which poses a huge challenge to feature extraction. 

Many studies have made great efforts in handcrafted feature extraction methods and feature reduction methods. However, conventional handcrafted methods still suffer from weaknesses in the following areas: (1) The handcrafted feature extraction and feature reduction methods need to be designed according to different kinds of monitored objects and signal sources, which depend on prior domain knowledge and expert experience [2]. As a result, these methods present low efficiency and poor generalization performance [5]. Especially when facing multi-sensor based condition monitoring tasks, due to the influence of noise and a large amount of redundant information, the feature engineering is more difficult and labor-intensive. It is difficult to select the suitable data fusion method and extract sparse features, which directly affects the performance MHM models [6]. (2) Considering that feature extraction and machine learning models that work in a cascaded way are independent of each other, without considering the relationship between them, so it is impossible to jointly optimize them [7]. The extracted features of input data determine the performance of the subsequent classification or prediction models [8], therefore, it is necessary to explore an effective method for multi-sensor time series feature extraction.

Deep learning (DL) [9] provides a powerful solution to above weaknesses. Unlike traditional models that are mostly based on handcrafted features, Deep neural network (DNN) can operate directly on raw data and learn features from a low level to a higher level to represent the distributed characteristics of data [10], which doesn’t require additional domain knowledge. After the layer-wise feature learning, DNN can adaptively extract deep and essential features according to the internal structure of massive data without domain knowledge. DNN has been successfully applied in speech recognition [11], image classification [12], motion recognition [13], text processing [14] and many other domains. In the past few years, the typical deep learning frameworks including deep autoencoder (DAE), deep belief network (DBN), deep convolutional neural network (DCNN), deep recurrent neural network (DRNN) and their variants have been developed in the field of machine health monitoring [15,16].

The unsupervised layer-by-layer pre-training process of DAE and DBN can reduce the need for labeled training samples and facilitate the training of DNN [15]. However, these fully-connected structures of DAE and DBN may lead to heavy computation cost and overfitting problems caused by huge model parameters, so DAE and DBN are not suitable for processing raw data, especially multi-sensor raw data.

The Convolutional neural network (CNN) is now the most prominent framework, which is usually used for learning spatial distribution of data. In the CNN model, the local connection mechanism between layers allows CNN to learn the local features of the data and the weight sharing mechanism can reduce model parameters. As a method to prevent overfitting, the spatial pooling layer of CNN can help the model learn more significant and robust features. The special structure of CNN can reduce the complication as well as the training time of the model. CNN has also been introduced to address time series data for mechanical fault diagnosis or remaining useful life estimation [17,18,19]. However, since the time series data is treated as static spatial data in CNN, where the sequential and temporal dependency are not taken into account, it may lead to the loss of most information between time steps [20].

The sensor data in the MHM system is usually a natural time series. Opposed to CNN, Long Short-Term Memory (LSTM) working in temporal domain is capable of sequence processing. As an advanced RNN variant, LSTM can adaptively capture long-term dependencies and nonlinear dynamics of time series data [21]. Although LSTM can directly receive raw data as input [20,22] and has been proven to be powerful for modeling time series data in MHM tasks, it does not take spatial correlation into consideration and easily leads to overfitting for multi-channel time series data containing crucial temporal and spatial dependencies.

Given the complementary strengths of CNN and LSTM, ConvLSTM is proposed for spatiotemporal sequence forecasting in [23]. Compared with LSTM, ConvLSTM preserves the spatial information [24], therefore it facilitates the spatiotemporal feature learning. Multi-sensor time series data in MHM tasks usually have high sampling rate (such as vibration signals and acoustic emission signals), so the 2D time series are sequential with long-term temporal dependency. The input sample always contains thousands of timestamps. The information of single timestamp may not be discriminative enough. Therefore, extracting local features in a short period of time can make it easier to learn long temporal dependencies between successive timestamps and often produce a better performance [7,25].

In this paper, a novel framework named Time-distributed ConvLSTM (TDConvLSTM) is proposed for intelligent MHM, which is powerful for learning spatiotemporal features of multi-sensor time series data on different time scales. TDConvLSTM is a hybrid end-to-end deep learning model, which has 5 main components: a data segmentation layer, time-distributed local spatiotemporal feature extractors, a holistic ConvLSTM layer, a fully-connected (FC) layer and a supervised learning layer. Firstly, the data segmentation layer utilizes a sliding window strategy along the temporal dimension to segment the normalized multi-sensor time series data into a collection of subsequences. Each subsequence is a 2D tensor and is taken as one time step in the holistic ConvLSTM layer. Then, all the subsequences are arranged in sequence and transformed into a 3D tensor. The local spatiotemporal feature extractor is applied to each time step to extract local spatiotemporal features inside a subsequence. The Holistic ConvLSTM layer can extract holistic spatiotemporal features between subsequences based on the local spatiotemporal features. Then a FC layer and a softmax or regression layer are stacked on the top of the model for classification or regression prediction. The main contributions of this paper are summarized as follows:

1. The ConvLSTM is first applied to extract spatiotemporal features of multi-sensor time series for real-time machine health monitoring tasks. It can learn both the complex temporal dependency and spatial dependency of multi-sensor time series, enabling the ConvLSTM to discover more hidden information than CNN and LSTM.
2. The time-distributed structure is proposed to learn both short-term and long-term features of time series. Therefore, it can make full use of information on different time scales.
3. The proposed end-to-end TDConvLSTM model directly works on raw time series data of multi-sensor and can automatically extract optimal discriminative features without any handcrafted features or expert experience. The time-distributed spatiotemporal feature learning method is not limited to a specific machine type or a fault type. Therefore, TDConvLSTM has wide applicability in MHM systems.
4. The proposed model is suitable for multisensory scenario and achieves better performance in both time series classification tasks and regression prediction tasks than some state-of-the-art models, which has been verified in the gearbox fault diagnosis experiment and the tool wear prediction experiment.

The remainder of the paper is organized as follows: In Section 2, machine health monitoring method based on CNN and LSTM are reviewed. In Section 3, the typical architecture of LSTM and ConvLSTM are briefly described. Section 4 illustrates the procedures of the proposed method. In Section 5, a gearbox fault diagnosis experiment and a tool wear prediction experiment are used to validate the effectiveness of the proposed method. Finally, conclusions are drawn in Section 6.

## 2. Related Work

### 2.1. Machine Health Monitoring Based on CNN

In some works, the raw sensor data in time domain has been transformed to frequency spectrum or time-frequency spectrum before being input to CNN models. The spectral energy maps of the acoustic emission signals are utilized as the input of CNN to automatically learn the optimal features for bearing fault diagnosis in [26]. Ding et al. proposed a deep CNN where wavelet packet energy images were used as input for spindle bearing fault diagnosis [27]. The methods presented above that indirectly processing time series data using CNN are time-consuming and limited by frequency domain and time-frequency domain transformation methods.

CNN can also directly address raw temporal signals in MHM tasks without any time-consuming preliminary frequency or time-frequency transformation. Zhang et al. presented a novel rolling element bearings fault diagnosis algorithm based on CNN, which performs all the operation on the raw temporal vibration signals without any other transformation [28]. Lee et al. addressed a CNN model for fault classification and diagnosis in semiconductor manufacturing processes with multivariate time-series data as the input [29]. In [19], CNN was first adopted as a regression approach for remaining useful life (RUL) estimation with multi-sensor raw data as model input. The raw time series data is treated as static spatial distribution data in CNN and its long temporal dependency information is lost, which makes CNN models perform poorly and error-prone. 

### 2.2. Machine Health Monitoring Based on LSTM

A LSTM based encoder-decoder scheme was proposed in [30] for anomaly detection, which can learn to reconstruct the “normal” time-series and thereafter the reconstruction error was used to detect anomalies. Based on the work in [30], an advanced LSTM encoder-decoder was proposed to obtain a health index in an unsupervised manner using multi-sensor time series data as input and thereafter the health index was used to learn a model for estimation of remaining useful life [31]. Bruin et al. utilized a LSTM network to timely detect faults in railway track circuits [32]. They compared the LSTM network with a convolutional network on the same task. It was concluded that the LSTM network outperforms the convolutional network for the track circuit case, while the convolutional networks are easier to train. Zhao et al. applied LSTM model encoded the raw sensory data into embedding and predicted the corresponding tool wear [22]. 

Due to the fact that multi-sensor time series data of mechanical equipment usually have high sampling rate, the input sequence may contain thousands of timestamps. Although the LSTM can directly work on raw time series data, the high dimensionality of input data will increase model size and make the model hard to train.

### 2.3. Hybrid Models Based on CNN and LSTM for Machine Health Monitoring

The hybrid models connecting CNN layers and LSTM layers in order, which expressed as CNN-LSTM in this paper, have been designed to extract both spatial and temporal features for speech recognition [33], emotion recognition in video [34] and gesture recognition [35] and so forth. A deep architecture was proposed for automatic stereotypical motor movements (SMM) detection by stacking an LSTM layer on top of the CNN architecture in [36]. Based on the work in [36], a further research that enhancing the performance of SMM detectors was presented in [37]. In the research, CNN was used for parameter transfer learning to enhance the detection rate on longitudinal data and ensemble learning was employed to combine multiple LSTM learners into a more robust SMM detector. In MHM tasks, the sensor data is often a multi-channel time series, which contains both temporal and spatial dependencies. The combination of CNN and LSTM has achieved higher performance on MHM tasks than single CNN and single LSTM [32]. Zhao et al. [38] designed a deep neural network structure named Convolutional Bi-directional Long Short-Term Memory networks (CBLSTM). One-layer CNN was applied in the model to extract local and discriminative features from raw input sequence, after which, two-layer bi-directional LSTMs were built on top of the previous CNN to encode the temporal information. The CBLSTM was able to outperform several state-of-the-art baseline methods in the tool wear estimation task. 

CNN-LSTM models usually learn spatial features first and thereafter learn temporal features. However, one layer in ConvLSTM can learn the temporal features and spatial features simultaneously by using convolutions operation to replace the matrix multiplication within the LSTM unit and pay more attention to how data changes between time steps. ConvLSTM has been used to extract spatiotemporal features of weather radar maps [23] and videos [39,40] but no application of ConvLSTM in MHM tasks has been found so far.

## 3. Introduction of ConvLSTM

### 3.1. Convolutional Operation

The convolutional layer and the activation layer are the most central parts of the CNN. Input data is first convoluted with the convolution kernel and the convolutional output is added with an offset. Then the following activation unit is used to generate the output features. The convolutional operation uses a local connected and weight shared method. Compared with traditional fully-connected layers, the convolutional layer can reduce model parameters and improve model calculation speed, which is more suitable for directly processing complex input data and extracting local features.

A convolutional layer usually contains multiple convolution kernels, that is, multiple filters. Assuming that the number of convolution kernels is *k*, each convolution kernel is used to extract one type of feature, corresponding to one feature matrix and *k* convolution kernels can output a total of *k* feature matrices. The convolutional operation can be expressed by:

(1) $$\;Z_{k}=f\left(W_{K}*X+b\right)$$

where X is the input data with size of m×n. $W_{K}$ is the Kth convolution kernel with size of $k_{1}\times k_{2}$. *b* denotes the offset. ‘∗’ denotes the convolution operator. The stride and the padding method in convolutional operation together determine the size of the Kth feature matrix $Z_{k}$. For example, when stride is (1,1) and using no padding during convolution, the size of $Z_{k}$ is $\left(m-k_{1}+1\right)\times \left(n-k_{2}+1\right)$. f is the nonlinear activation function which performs nonlinear transformation on the output of the convolutional layer. The commonly used activation functions are sigmoid, tanh and ReLu.

### 3.2. From LSTM to ConvLSTM 

**Figure 1:**
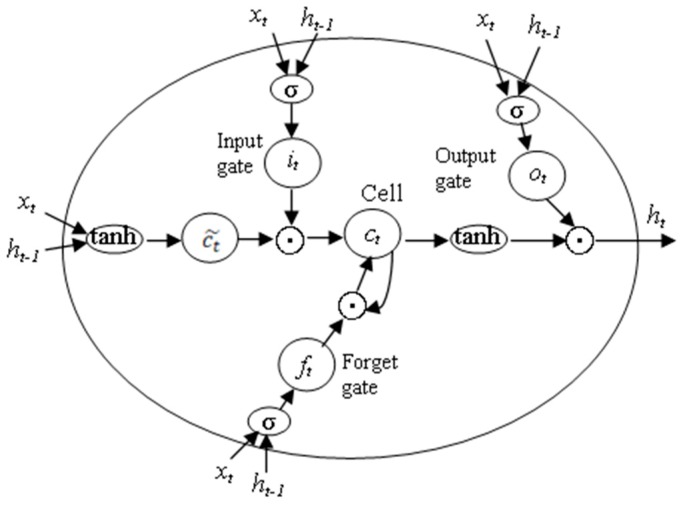
The structure of three gates in the Long Short-Term Memory (LSTM) cell.

LSTM has been proven to be the most stable and powerful model to learn long-range temporal dependences in practical applications as compared to standard RNNs or other variants. The structure of the repeating module in the LSTM is shown in Figure 1. The LSTM uses three ‘gate’ structures to control the status of the memory cell $c_{t}$. The three gates have the ability to remove or add information to the cell state. The three gates are input gate $i_{t}$, forget gate $f_{t}$ and output gate $o_{t}$, which can be understood as a way to optionally allow information to pass through [41]. The process of information passing and updating in LSTM can be described by the equations shown in (2)–(7), where ‘◦’ denotes the Hadamard product. At each time step t, the memory cell state $c_{t}\;$and the hidden state $h_{t}\;$can be update by the current input $x_{t}$, the hidden state at previous time step $h_{t-1}$ and the memory cell state at previous time step $c_{t-1}$. When a new input comes, $f_{t}$ can decide how many information in $c_{t-1}\;$ should be forgotten. Then, $i_{t}\;$and $\tilde{c_{t}}$ will decide what new information can be store in the cell state. The next step is to update the old cell state $c_{t-1}$ into the new cell state $c_{t}$. Finally, $x_{t}$, $h_{t-1}$ and $c_{t}\;$ determine the output $\;h_{t}$. The input, cell state and output of the LSTM are all 1D vectors. The LSTM uses full connections in input-to-state and state-to-state transitions.

(2) $$\;f_{t}=\sigma \left(W_{xf\;}x_{t}+W_{hf}h_{t-1}+b_{f}\right)$$

(3) $$\;i_{t}=\sigma \left(W_{xi}x_{t}+W_{hi}h_{t-1}+bi\right)$$

(4) $$\tilde{c_{t}}=tanh\left(W_{xc}x_{t}+W_{hc}h_{t-1}+b_{c}\right)$$

(5) $$\;c_{t}=f_{t}◦c_{t-1\;}+i_{t}◦\tilde{c_{t}}$$

(6) $$\;o_{t}=\sigma \left(W_{xo\;}x_{t}+W_{ho}h_{t-1}+bo\right)$$

(7) $$\;h_{t}=o_{t}◦tanh\left(c_{t}\;\right)$$

LSTM is capable of modeling time series data with long-term dependency in MHM tasks. Although LSTM can also be applied on multi-dimensional sequence by reshaping the multi-dimensional input to a 1D vector but it fails to maintain structural locality [39] and contains too much redundancy [23].

To exploit both spatial and temporal information in multi-sensor time series data, we proposed a model based on ConvLSTM. ConvLSTM is an extension of LSTM, which replaces the matrix multiplication in LSTM with convolutional operation [23]. The equations of ConvLSTM are shown in (8)–(13), where ‘∗’ denotes the convolution operator. The input $x_{t}\;$, cell state $c_{t}$ and hidden output ${\;h}_{t}\;$are all 3D tensors, where the first two dimensions are spatiotemporal information and the last dimension is the number of convolutional filters. The convolutional operation of ConvLSTM can reduce the number of model parameters and prevent overfitting [42]. ConvLSTM retains the advantages of learning temporal dependency between different time steps, in addition to this, it can capture the local spatial information. Therefore, ConvLSTM can learn more discriminative features from multi-sensor time series data.

(8) $$\;f_{t}=\sigma \left(W_{xf}*x_{t}+W_{hf}*h_{t-1}+b_{f}\right)\;$$

(9) $$\;i_{t}=\sigma \left(W_{xi}*x_{t}+W_{hi}*h_{t-1}+bi\right)$$

(10) $$\tilde{c_{t}}=tanh\left(W_{xc}*x_{t}+W_{hc}*h_{t-1}+b_{c}\right)$$

(11) $$\;c_{t}=f_{t}◦c_{t-1\;}+i_{t}◦\tilde{c_{t}}$$

(12) $$\;o_{t}=\sigma \left(W_{xo\;}*x_{t}+W_{ho}*h_{t-1}+bo\right)\;\;$$

(13) $$\;h_{t}=o_{t}◦tanh\left(c_{t}\;\right)$$

## 4. Methods

### 4.1. Notation

In the multisensory MHM scenario, the time series collected from monitored machine is a sequence of real-valued data points generated by M different sensor channels. The input sample of the model can be represented as a 2D matrix, which is denoted as $X=\left\{x_{1},x_{2},\cdots ,x_{L}\right\}$, where L is the length of the sample and the input data $x_{i}$ at the ith timestamp is a vector with M elements. Each training sample has a corresponding target value Y. Y is a categorical value that has been encoded to a one-hot vector in the fault classification task or a real-valued data in the regression prediction task. The machine health monitoring task is defined to obtain the target value Y based on multi-sensor time series data X. In the following text, we divide X into N local subsequences, then, the input can be denoted as $X=\left\{P_{T1},P_{T2},\cdots ,P_{TN}\right\}$, each subsequence $P_{Ti}\in R^{M\times l}$ is denoted as $P_{Ti}=\left\{x_{Ti}^{1},x_{Ti}^{2},\cdots ,x_{Ti}^{l}\right\}$, where $x_{Ti}^{k}\in R^{M}$ is the kth timestamp in the ith subsequence. l is the length of each subsequence. Further, (A,B) represents the shape of a tensor with A rows and B columns. 

### 4.2. The Proposed TDConvLSTM Model

**Figure 2:**
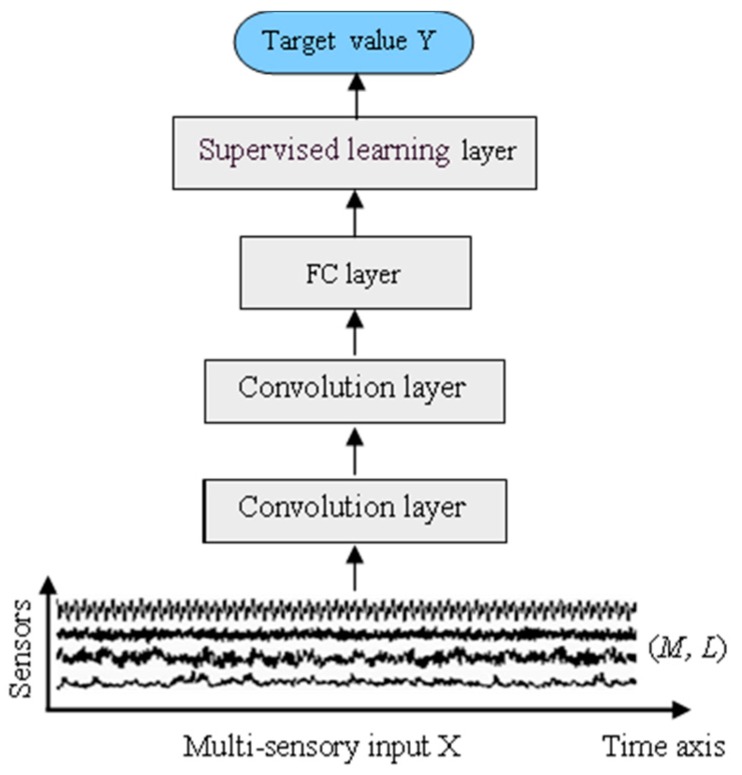
The framework of the basic ConvLSTM model.

In this section, a time-distributed ConvLSTM model (TDConvLSTM) is presented for multi-sensor time series based machine health monitoring. TDConvLSTM is a hybrid end-to-end framework that focuses on time-distributed spatiotemporal feature learning which is an extension method of basic ConvLSTM. The basic ConvLSTM model are consists of only a few ConvLSTM layers, a FC layer and a supervised learning layer, which is shown in Figure 2. ConvLSTM directly extract spatiotemporal features in the whole range of the multi-sensory input data. Although ConvLSTM can directly work on multi-sensor time series data to simultaneously capture the temporal dependencies and spatial dependencies, the input time series in the MHM task always contains thousands of timestamps, which will make the model size too large and make it difficult to train the model. The basic ConvLSTM model cannot learn long temporal dependencies well. Therefore, extracting local features in a local range of the input data before extracting features in the whole range can make it easier to learn long temporal dependencies between successive timestamps and promote the model for better performance.

**Figure 3:**
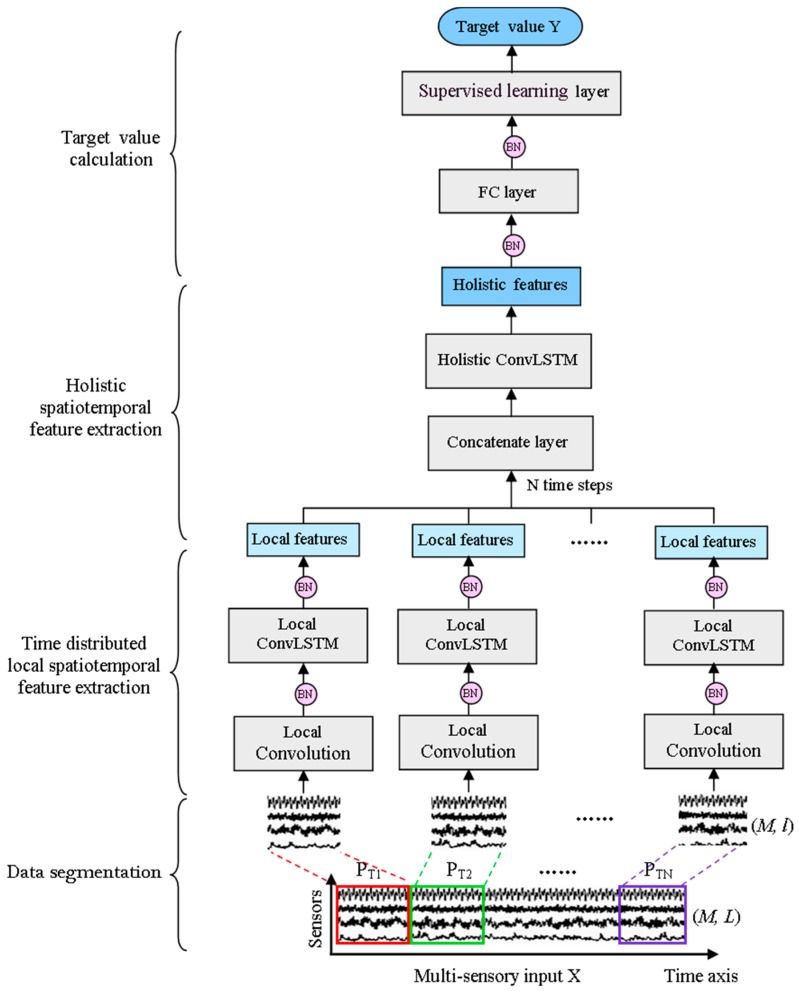
The framework of the proposed TDConvLSTM model (TDConvLSTM).

Considering the above shortcomings of basic ConvLSTM, a time-distributed ConvLSTM model (TDConvLSTM) is proposed. The proposed TDConvLSTM has three major procedures: data segmentation, time-distributed local spatiotemporal features extraction and holistic spatiotemporal features extraction. The framework of the TDConvLSTM model is shown in Figure 3. Firstly, the normalized multi-sensor time series is segmented into a collection of subsequences using a sliding window along the time dimension. Then all the subsequences are reorganized into the shape that fit into the subsequent time-distributed local spatiotemporal feature extraction layers. Holistic ConvLSTM layers stacked on the top of time-distributed local spatiotemporal feature extraction layers are used to extract holistic spatiotemporal features between subsequences based on the time-distributed local spatiotemporal features. At last, a FC layer and a supervised learning layer are stacked on the top of the model to obtain the target value Y. The local spatiotemporal features extracted in each subsequence only contain the features of a part of the input data. The holistic features are extracted from local features of all subsequences to learn the long temporal spatiotemporal dependencies between subsequences. So, the holistic features contain the spatiotemporal features of the whole input data. Local spatiotemporal features are extracted before extracting holistic spatiotemporal features, which can make it easier to learn long temporal dependencies between successive timestamps and enable the TDConvLSTM to get better performance.

#### 4.2.1. Data Normalization and Segmentation 

Each channel of the multi-sensor time series data may come from different kinds of sensors and the order of magnitude of each channel may be different. If the raw multi-sensor time series data is used directly to train the model, the model will be difficult to converge. Therefore, the raw multi-sensor time series data is normalized by the z-score method before being input to the model. The main purpose of z-score is to convert data of different magnitudes into the same order of magnitude to ensure the comparability between the data. The conversion function can be expressed as:

(14) $$\;x_{j}^{z}=\frac{x_{j}-\mu_{j}\;}{\sigma_{j}}\;$$

where, $x_{j}\;$ is the time series of the jth sensor channel. $\mu_{j}\;$ and $\sigma_{j}$ are the mean and standard deviation of $x_{j}$. $x_{j}^{z}$ is the time series data after z-score normalization.

The input of the model $X\in R^{M\times L}$ is segmented into N local subsequences by a sliding window along the time dimension. All subsequences have the same length l *and* $N=\frac{L}{l}$. Setting l to a value that makes L divisible by l is more appropriate. If L cannot be divisible by l, the remainder part will be discarded. In other words, just the integer part of N will be retained. The demonstration of data segmentation is shown in Figure 3. Each subsequence $P_{Ti}\in R^{M\times l}$ is a window of the multi-sensor input signal and is regarded as one-time step in the holistic ConvLSTM layer.

The length l of each subsequence is a hyperparameter of the TDConvLSTM model, which controls the number of subsequences, that is, the number of time steps in the holistic ConvLSTM layer. It is obvious that a small l may not be able to obtain discriminative local features. Oppositely, if l is large, the number of time steps of the holistic ConvLSTM layer will be decreased, so that much holistic spatiotemporal information will be lost. A suitable l can be selected by comparing experiments.

#### 4.2.2. Time-Distributed Local Spatiotemporal Feature Extraction

After data segmentation, a local feature extractor is used to extract spatiotemporal features of each subsequence. The local feature extractor is applied to each subsequence $P_{Ti}$ simultaneously using a “TimeDistributed wrapper,” which is shown in Figure 3. *N* local feature extraction processes are performed simultaneously and independently of each other.

Since local feature extractors of different time steps have the same structure, we focus on one local feature extractor with the input subsequence $P_{Ti}=\left\{x_{Ti}^{1},x_{Ti}^{2},\cdots ,x_{Ti}^{l}\right\}$, which is a (l×M) tensor.$\;P_{Ti}$ is divided into n slices, each slice $f_{q},\left(q=1,2,\cdots n\right)$ is a $\left(l_{0}\times M\right)$ tensor, where $l_{0}=\frac{l}{n}$. As a result, the input $P_{Ti}$ is transformed into a 3D tensor with shape of $\left(n,M,l_{0}\right)$. We can think of the 3D tensor as a movie and $f_{q}$ is a frame in the movie. Then the 3D tensor will be input to the local feature extractor. 

**Figure 4:**
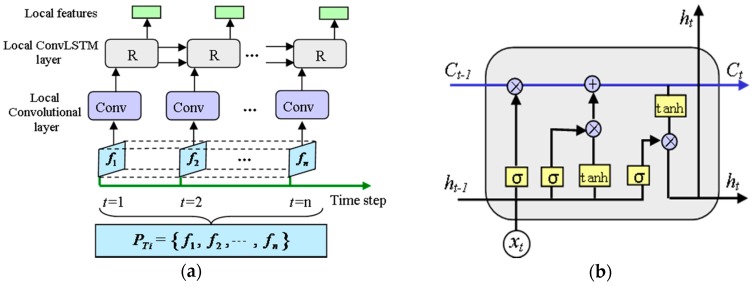
Local spatiotemporal feature extractor: (**a**) Structure of the local spatiotemporal feature extractor; (**b**) Diagram of the recurrent cell in ConvLSTM.

The local feature extractor consists of a time-distributed local convolutional layer and a local ConvLSTM layer, which is shown in Figure 4. There are two types of features embedded in $P_{Ti}$, that is, temporal features inside a sensor channel and spatial features between different sensor channels. We applied the convolutional operation to each frame $f_{q}$ simultaneously using a local “TimeDistributed wrapper.” 2D kernels with shape $\left(k_{1},1\right)$ are applied in the first local convolutional layer to extract features inside a sensor channel and preserve the independence of each channel. In addition, we choose a larger convolution stride $\left(k_{1},1\right)$ than conventional (1,1) used in image recognition. The large convolutional stride can reduce the dimensionality of the input data and keep the timing unchanged. We apply c filter channels to each of the first three layers, which enable the model to get more non-linear functions and learn more information of the current sequence. The local convolutional layer returns a feature with shape of $\left(n,l_{1},M,c\right)$, which is thereafter served as the input of the local ConvLSTM layer. In the local ConvLSTM layer, 2D kernels with shape $\left(k_{2},M\right)$ and convolutional stride (1,1) are adopted to learn deeper temporal features and the dependencies between different sensor channels. The local ConvLSTM layer returns a feature $Lf_{Ti}$ with shape of $\left(n,l_{2},c\right)$.

After two local feature extraction layers, local spatiotemporal features inside each subsequence $P_{Ti}$ are extracted and the noise of the raw input data is eliminated. The time-distributed local feature extractors turn the raw input time series into a shorter sequence, which make it easier to learn long temporal dependencies.

#### 4.2.3. Holistic Spatiotemporal Feature Extraction

After *N* time-distributed local spatiotemporal feature extractors, a local feature sequence with *N* time steps is returned with shape of (*N*, *n*, *l*_2_, *c*). A holistic ConvLSTM layer is applied on the local feature sequence to extract holistic spatiotemporal features. There are *N* time steps in the holistic ConvLSTM layer and the local feature *Lf_Ti_* = (*n*, *l*_2_, *c*) is the input at time step Ti. Small 2D kernels $\left(k_{3},k_{3}\right)$ and convolution stride (1,1) are adopted to further learn deeper and sparser spatiotemporal features. After holistic spatiotemporal feature extraction, holistic spatiotemporal feature at each time step is flattened into a 1D tensor, whose length is $l_{3}$. Then spatiotemporal features of N time steps are concatenated into a 1D feature vector v with length of $N\times l_{3}$.

#### 4.2.4. Supervised Learning Layer

At last, the feature vector ***v*** is passed into another FC layer and a supervised learning layer. If the targets are discrete labels such as fault types, the supervised learning layer is a softmax layer, which is defined as:

(15) $$\;P\left(y=j\;\right)=\frac{e^{\theta_{j}^{T}v}}{\sum_{k=1}^{K}e^{\theta_{j}^{T}v}}\;$$

where K is the number of labels and θ denotes parameters of softmax layer.

If the targets are continuous values such as remaining useful life (RUL) and tool wear, the supervised learning layer can be a linear-regression layer given by:

(16)  y=Wv+b   

where W and b denote the transformation matrix and the offset in the linear regression layer. 

The error between predicted values and truth values in training data can be calculated and back propagated to train the parameters of the whole model. Then, the trained model can be applied to monitor machine health condition.

#### 4.2.5. Batch Normalization

The proposed model has a multi-layer structure. The model parameters change continuously in the training process, resulting in continuous changes in the input distribution of each subsequent layer. The learning process has to adapt each layer to the new input distribution, so the learning rate has to be reduced, resulting in a slow model converge rate. The batch normalization (BN) layer [43] is designed to reduce the shift of internal covariance and accelerate the training process of deep model by normalizing the output of each layer to obey the normal distribution. In our model, BN layers are added right after the local convolutional layer, the local ConvLSTM layer and the FC layer and before the activation unit. Assume that the input vector of the BN layer is x, $x\in R^{m}$, then the output of the BN layer can be calculated by:

(17) $$\;y_{i}=\gamma x_{i}^{\prime }+\beta$$

(18) $$\;x_{i}^{\prime }=\frac{x_{i}-\mu_{B}\;}{\sqrt{\sigma_{B}^{2}+\epsilon }}$$

(19) $$\;u_{B}=\frac{1}{m}\sum_{i=1\;}^{m}x_{i}$$

(20) $$\;\sigma_{B}^{2}=\frac{1}{m}\sum_{i=1\;}^{m}{\left(x_{i}-\mu_{B}\right)}^{2}\;$$

where $\mu_{B}$ is the mean of $x_{i}$, $\sigma_{B}^{2}$ is the variance of $x_{i}$, ϵ is a small constant, γ and β are parameters that need to be learned in the model. BN can accelerate the convergence of the model and prevent overfitting. With BN layers, we can reduce the use of Dropout and adopt a large learning rate.

## 5. Experiments and Discussion

To verify the effectiveness of our proposed TDConvLSTM model, two experiments about gearbox fault diagnosis and real industrial milling tool wear monitoring were conducted.

### 5.1. Case Study 1: Gearbox Fault Diagnosis 

#### 5.1.1. Data Collection

**Figure 5:**
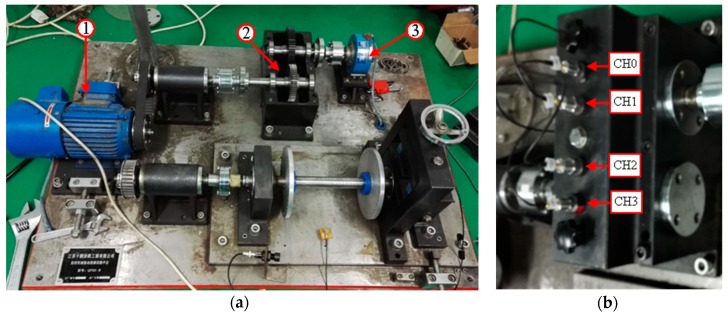
Gearbox test rig: (**a**) Main units: (1) Motor (2) Parallel gearbox (3) Magnetic powder brake (**b**) Locations of sensors.

To verify the effectiveness of the proposed TDConvLSTM model for gearbox fault diagnosis, an experiment was conducted on a gearbox test rig as shown in Figure 5a. The gearbox test rig is composed of three main units including the motor, the parallel gearbox and the magnetic powder brake. Four single-axis accelerometers were mounted vertically on the upper surface of the gearbox. Figure 5b shows the locations of sensors. The gearbox test rig has been operated under 4 different health conditions. The descriptions of different health conditions are listed in Table 1. Gearbox in each health condition has been operated at three speeds (280 rpm, 860 rpm and 1450 rpm) of the pinion. Vibration signals of 4 channels under each speed were acquired synchronously through a data acquisition box. The sampling frequency was 10.24 kHz and the sample time was 5s.

The acquired signals are first normalized as described in Section 4.2.1. Then, a sliding window with length of 5120 is used to slice the signals with overlap. There are 450 samples for each health condition under each identical operating speed. According to the operating speeds, all samples are grouped into four datasets (D1–D4) to test the performance of the proposed model respectively. D1, D2 and D3 contain samples at speeds of 280 rpm, 860 rpm and 1450 rpm, respectively. D1, D2 and D3 are brought together to form dataset D4. The samples at the three rotational speeds for each health condition are taken together as the same class in D4. There are 1800 samples in D1, D2 and D3, respectively. We first shuffle the sample order of D1, then, two-thirds of the 1800 samples are selected as the training dataset and the remaining one-third samples are selected as the testing dataset. The same processing procedure is used for D2 and D3. Finally, there are 1200 training samples and 600 testing samples in dataset D1, D2 and D3, respectively. There are 3600 training samples and 1800 testing samples in dataset D4.

#### 5.1.2. Parameters of the Proposed TDConvLSTM

The architecture of the proposed TDConvLSTM model used in experiments is built according the procedures described in Section 4. It should be noted that the hyperparameters of the model are selected through cross-validated experiments. The hyperparameters such as the kernels, strides and channels in main layers with the best performance are displayed in Table 2. Batch normalization is used right after each main layer to improve the performance of the model. The batch normalization axis is set to the channel axis. The activation function of the last layer is softmax and activation functions of other layers are all set to sigmoid. The categorical cross-entropy is adopted as the loss function and Adam is employed for model training. The dropout rate is set to 0.2. 

**Figure 6:**
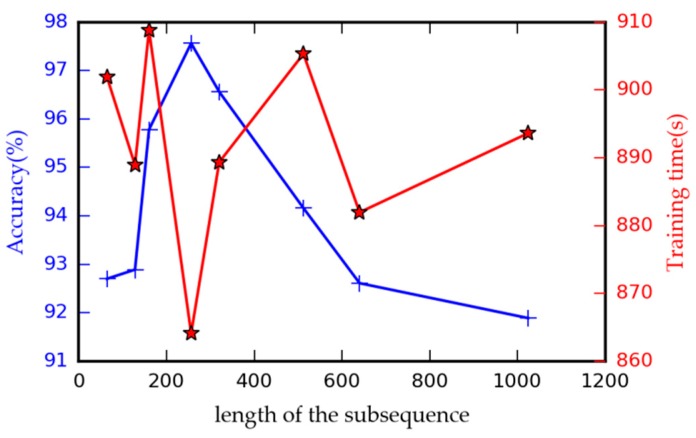
Accuracy and training time under different length of the subsequence.

As stated in Section 4.2.1, the length of subsequence l is a crucial hyperparameter of the TDConvLSTM model, so it is meaningful to research the influence of different l on the performance of the proposed model. In this research, we set l to 64, 128, 160, 256, 320, 512, 640 and 1024 respectively to test the performance of the model. Each subsequence $P_{Ti}$ in the test model is divided into 8 slices. The other parameters of the model are same as shown before. An appropriate l is needed to fit the signals under different operating conditions, so the dataset acquired under nonstationary condition is suitable to test the performance of the model with different l. Dataset D4 is used to train the model for 15 epochs with the batch size of 20. The fault classification accuracy and the model training time are used to evaluate the model performance. The performances of the model with different l are compared and shown in Figure 6. It can be seen that when the length of the subsequence l is set to 256, the proposed model has the best performance in both fault classification accuracy and model training speed. Smaller and larger l would decline the performance of the proposed model. The model with a small l cannot learn discriminative local features. A large l would decrease the time steps of the holistic ConvLSTM layer, as a result, the model cannot obtain effective holistic spatiotemporal information. Therefore, l is set to 256 in the following experiments.

#### 5.1.3. Results and Discussion

To prove the advantage of the proposed TDConvLSTM, the same multi-sensor data is processed by some comparative models: empirical mode decomposition and support vector machine method (EMD-SVM), convolutional neural network (CNN), long short-term memory neural network (LSTM), a hybrid model that series connects CNN and LSTM (CNN-LSTM) and the proposed TDConvLSTM model without batch normalization (TDConvLSTM without BN). 

To compare the performance of traditional machine learning models based on handcrafted features with the deep learning models based on raw sensor data, EMD-SVM is adopted as a comparative model. In EMD-SVM, the data of each sensor channel is decomposed by EMD firstly and the normalized energy, kurtosis, kurtosis and variance of the top five intrinsic mode functions are extracted as handcrafted features. A total of 80 features are obtained from four sensor channels to constitute a feature vector, which is used as the input of the SVM.

It should be noted that, all the deep learning models in this experiment are consist of five main layers. The last two layers in each model are a FC layer with size of [100] with dropout and a softmax layer with size of [4]. In CNN, three pairs of convolutional layers and pooling layers are stacked. The filter size, stride, channel and pooling size of three pairs of layers are set to [(4, 1), (4, 1), 10, (2, 1)], [(1, 4), (1, 1), 10, (2, 1)] and [(2, 1), (1, 1), 10, (2, 1)] respectively. In LSTM, the raw data with size of (5120, 4) is divided into 20 time steps firstly. Each time step is a 2D tensor with size of (256, 4). Then we flatten the 2D tensor into a 1D tensor (1024). As a result, the raw data is reshaped to (20, 1024). For the LSTM model, three LSTM layers with sizes of [500], [100] and [10] are stacked. In the CNN-LSTM model, two CNN layers with size of [(4, 1), (4, 1), 10] and [(1, 4), (1, 1), 10] are firstly designed, which is followed by a LSTM layer with size of [50]. Between the two CNN layers, a pooling layer with size of (2, 1) is adopted. Except the softmax layer, the activation function of each layer in CNN model and CNN-LSTM model are set to ReLu and activation functions in LSTM model are set to sigmoid. Parameters of the proposed TDConvLSTM are shown in Section 5.1.2. The Parameter settings of the TDConvLSTM without BN keep the same as the proposed TDConvLSTM model, except that all the batch normalization layers are removed. Dataset D1, D2, D3 and D4 are used to test all the comparative models respectively. The testing results are listed in Table 3. It is shown that our proposed TDConvLSTM model can diagnose the faults of the gearbox effectively with the highest test accuracy both under constant rotation speed and nonstationary rotation speed. 

**Figure 7:**
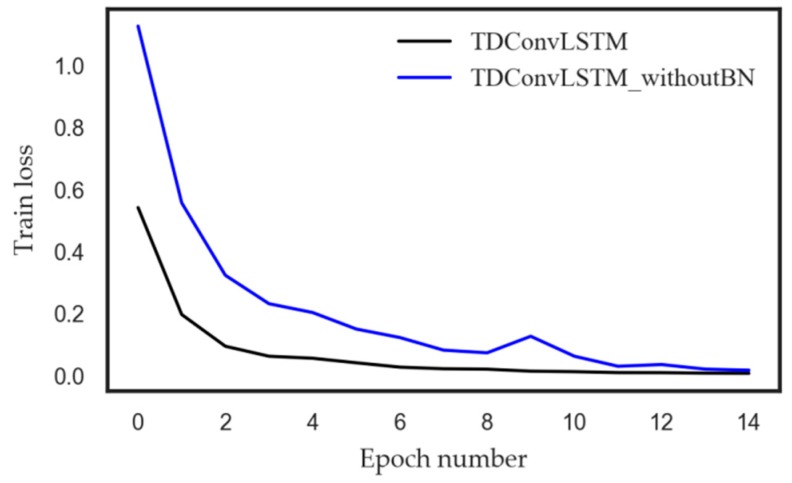
The effect of batch normalization (BN) on model training.

As shown in Table 3, all the deep learning models based on raw sensor data can achieve better performance than the handcrafted features based model EMD-SVM. Under constant rotation speed, the proposed TDConvLSTM model can achieve higher test accuracy than CNN and LSTM, which can be explained that the proposed TDConvLSTM model can both extract temporal features and spatial features of multi-sensor time series, which enables the TDConvLSTM layer to discover more hidden information than CNN and LSTM. The ConvLSTM structure can simultaneously learn the temporal features and spatial features and pay more attention to how data changes between time steps, so it can obtain better performance that CNN-LSTM structure. Under nonstationary rotation speed, the features that related to faults are hidden on different time scales. The proposed time-distributed structure can learn both short-term and long-term spatiotemporal features of time series, that is, it can make full use of the information on different time scales in the signal. Therefore, the proposed model can deliver better performance under nonstationary rotation speed. The comparison of TDConvLSTM and TDConvLSTM without BN proves that BN can improve the fault diagnose accuracy of the TDConvLSTM model. In addition to this, BN can improve the speed of model convergence, which is corroborated in Figure 7. The time required to calculate each sample is just 0.006s with i5-4570 CPU, which proves that the proposed TDConvLSTM model can be used for real-time mechanical fault diagnosis.

#### 5.1.4. Feature Visualization

**Figure 8:**
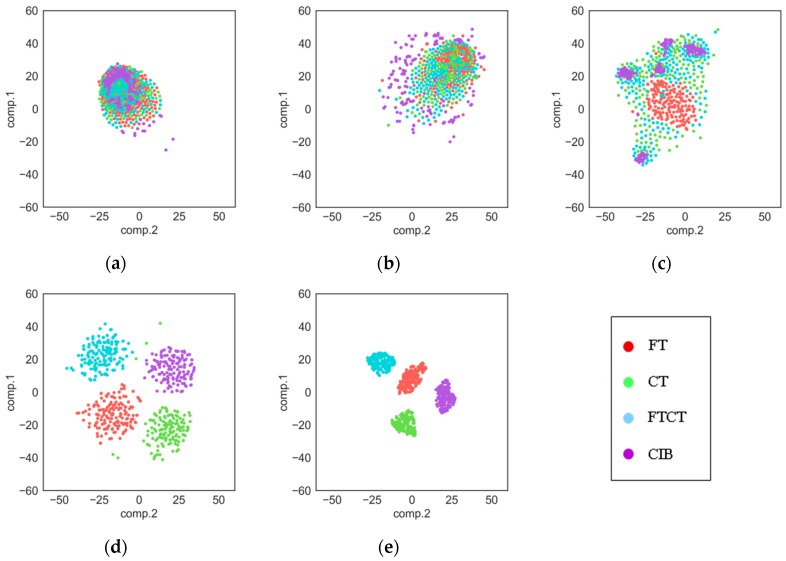
Feature visualization: (**a**) Raw data; (**b**) Local Convolutional layer; (**c**) Local ConvLSTM layer; (**d**) Holistic ConvLSTM layer; (**e**) Fully-connected (FC) layer.

As we know, deep learning models work like a black box, so it is hard to understand its process of extracting features. In this section, the t-SNE method [44] is used to show the features extracted by each layer in our proposed TDConvLSTM model. T-SNE is an effective dimensionality reduction method, which can help us to visualize high-dimensional data by mapping the data from high-dimensional space to a two-dimensional space. Features extracted by each layer are respectively converted to a two-dimensional feature map. The feature maps of raw data, the local convolutional layer, the local ConvLSTM layer, the holistic ConvLSTM layer and the FC layer are shown in Figure 8, in which features of different fault types are distinguished by different colors. It can be seen that as the layers get deeper and deeper, the features of different fault types become more and more separate. As shown in the Figure 8a, raw data of four fault types are all mix together. Then the local convolutional layer disperses all features, which is shown in Figure 8b. Starting from the local ConvLSTM layer, the features of the same fault type begin to cluster. In the Figure 8c, we can see that the FT type and the CIB type start clustering first, while the CT type and FTCT type are still mix together. It is because that both the CT type and FTCT type have a root crack tooth in the big gear, so they have some same features. As we can see in Figure 8d, after the local ConvLSTM layer, the features of four fault types are almost separated. At last, the FC layer further separates the features of the four fault types and further clusters the features of the same fault type.

### 5.2. Case Study 2: Tool Wear Monitoring

#### 5.2.1. Experiment Setup and Data Description

**Figure 9:**
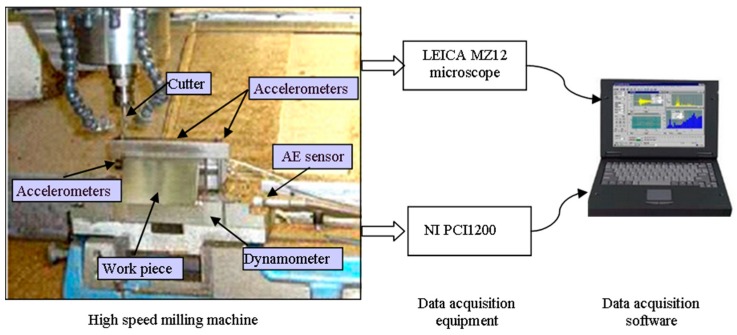
The experiment setup for tool wear monitoring

The experiment was implemented on a high-speed CNC machine with a spindle speed of 10,400 rpm [45]. The experiment setup is illustrated in Figure 9. The material of the work-piece is Inconel 718. Ball-nose cutting tools of tungsten carbide with 3 flutes were used to mill the work-piece. The operation parameters are as follows: the feed rate in the x direction was 1555 mm/min; the depth of cut in the y direction (radial) was 0.125 mm; the depth of cut in the z direction (axial) was 0.2 mm. During the milling process, a Kistler quartz 3-component platform dynamometer, three Kistler Piezo accelerometers and a Kistler acoustic emission (AE) sensor were used to measure the cutting force, machine tool vibration and the high frequency stress wave generated by the cutting process, respectively. Seven channels of signals (force_x, force_y, force_z, vibration_x, vibration_y, vibration_z, AE_RMS) were acquired by DAQ NI PCI1200 with a sampling frequency of 50 kHz. After completing one surface milling, which was regarded as one cut, the corresponding flank wear of the three flutes were measured offline using a LEICA MZ12 microscope. The tool wear is the average wear of the three flutes. There are 300 cuts in each tool life and the multi-sensor data of one cut is regarded as a sample. The target value of a sample is the corresponding tool wear. Finally, three tool life dataset C1, C4 and C6 are selected to test our model. Due to the high sampling rate of raw data, the length of each sample is up to 200 thousand. The raw data is first downsampled; as a result, the length of each sample is reduced to 20,000. The acquired signals are first normalized as described in Section 4.2.1. In each testing case, two of the datasets are selected as the training dataset and the other one is used as the testing dataset. There are three model testing cases and according to the testing dataset, they are marked as C1, C4 and C6, respectively.

#### 5.2.2. Model Settings

For our proposed TDConvLSTM model tested in tool wear monitoring experiments, the main parameters in main layers are listed in Table 4. The subsequence length *l* of the proposed model in this experiment is set to 500. The mean squared error is adopted as the loss function and the Nesterov-accelerated adaptive moment estimation (Nadam) algorithm [46] is employed as the optimizer for model training. It should be noted that, we formulate the tool wear prediction task as a regression prediction problem, so the supervised learning layer is a linear-regression layer and the activation in this layer is set to linear. The dropout rate of the FC layer is set to 0.5. Other parameters keep the same as stated in Section 5.1.2.

#### 5.2.3. Results and Discussion

Three models including CNN, LSTM and CNN-LSTM are compared with the proposed model. Their structures are same as that described in Section 5.1.3, except that some parameter settings are changed. In CNN, the filter size, stride, filter number and pooling size of three pairs of convolutional and pooling layers are set to [(500, 3), (250, 3), 10, (2, 1)], [(4, 2), (1, 1), 10, (2, 1)] and [(2, 1), (1, 1), 10, (2, 1)], respectively. The activation functions of CNN models are all set to ReLu. In LSTM, the raw data with size of (20,000, 7) is divided into 40 time steps along the temporal dimension firstly. The data of each time step is reshaped to a 1D tensor and the raw data finally reshaped to (40, 3500). The output size of the three LSTM layers are [1000], [100] and [10] respectively. All the activation functions in LSTM model are set to tanh. In the CNN-LSTM model, the filter size, stride, filter number of two CNN layers are set to [(500, 3), (250, 3), 10] and [(500, 3), (1, 1), 10], respectively. The pooling size in the pooling layer is (2, 1). The size of the LSTM layer is set to [10]. The settings of the last two layers of the three models are same as the TDConvLSTM model described in Section 5.2.2. 

Mean absolute error (MAE) and root mean squared error (RMSE) of the true targets and the predicted targets are adopted as the indicators of model performance. The corresponding equations for the calculations of MAE and RMSE are given as follows:

(21) $$\;MAE=\frac{1}{n}\sum_{i=1\;}^{n}\left|y\_test-y\_pre\right|$$

(22) $$\;RMSE=\sqrt{\frac{1}{n}\sum_{i=1\;}^{n}{\left(y\_test-y\_pre\right)}^{2}}$$

where *y_test* is the true tool wear value in the test dataset and *y_pre* is the predicted tool wear value, n is the number of testing samples.

MAE and RMSE of all models in three different model testing cases are shown in Table 5. As we can see in the table, the TDConvLSTM model and the CNN-LSTM model both perform better than CNN and LSTM. The result can be explained that the TDConvLSTM model and the CNN-LSTM model can extract spatiotemporal features, while, the CNN model discards the long-term temporal correlation information in each channel data and the LSTM model discards the spatial correlation information between different channels. The hybrid models can discover more hidden information than CNN and LSTM. 

**Figure 10:**
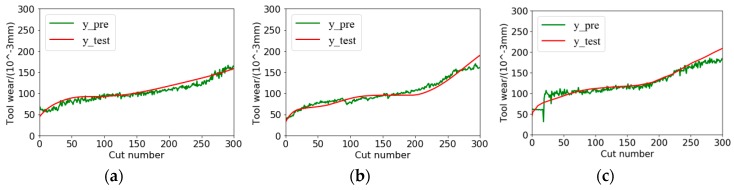
Regression performances of TDConvLSTM for three different testing cases: (**a**) C1; (**b**) C4; (**c**) C6.

It is shown that our proposed TDConvLSTM model achieves the best performance among all compared models. The most competitive CNN-LSTM model independently extracts the spatial features and the temporal features in succession, while, the TDConvLSTM model can simultaneously learn the temporal features and spatial features and pay more attention to capture the data changing features between time steps. The time-distributed structure can prompt the TDConvLSTM model make full use of information on different time scales. The above two advantages make the proposed model get better performance. The regression performances of TDConvLSTM in three different testing cases are illustrated in Figure 10. It is found that the predicted tool wear values are able to follow the trend of true tool wear values well with very small error. The testing time for each sample is 0.013s with i5-4570 CPU, which proves that the proposed TDConvLSTM model can be used for real-time tool wear monitoring.

## 6. Conclusions

The TDConvLSTM model has been proposed in this paper to extract spatiotemporal features of multi-sensor time series for machine health monitoring. The TDConvLSTM model is suitable for raw multi-sensor data and does not require any expert knowledge and feature engineering. In TDConvLSTM, the normalized multi-sensor time series is first segmented into a collection of subsequences using a sliding window. Then a time-distributed local feature extractor is designed with a time-distributed convolution layer and a ConvLSTM layer, which is employed in each subsequence to extract local spatiotemporal features inside a subsequence. The holistic ConvLSTM layer that stacked on the top of time-distributed local feature extractors can extract holistic spatiotemporal features between subsequences. At last, the fully-connected layer and the supervised learning layer can further reduce the feature dimension and obtain the machine health condition. The time-distributed structure can learn both short-term and long-term spatiotemporal features of multi-sensor time series. Therefore, it can make full use of information on different time scales. In the gearbox fault diagnosis experiment and the tool wear monitoring experiment, the results have confirmed the superior performance of the proposed TDConvLSTM model.

In future work, we plan to apply the proposed time-distributed spatiotemporal feature learning method in machine remaining useful life prediction tasks and continue to optimize our model to get better performance.

## Figures and Tables

**Table 1:** Description of four gearbox health conditions.

Label	Condition	Description	Speed (rpm)
0	FT	A root fracture tooth in the big gear	280, 860 and 1450
1	CT	A root crack tooth in the big gear	280, 860 and 1450
2	CTFT	A root crack tooth in the big gear and a half fracture tooth in the small gear	280, 860 and 1450
3	CIB	A crack on the inner race of the bearing	280, 860 and 1450

**Table 2:** Parameters of the proposed model used in gearbox fault diagnosis experiments.

No.	Layer Type	Kernel	Stride	Channel	BN Axis	Activation
1	Local Convolution	(4,1)	(4,1)	4	4	sigmoid
2	Local ConvLSTM	(1,4)	(1,1)	4	5	tanh
3	Holistic ConvLSTM	(2,2)	(1,1)	4	4	tanh
4	FC layer	100	-	1	−1	sigmoid
5	Supervised learning layer	4	-	1	-	softmax

**Table 3:** Testing accuracy of comparative methods.

Model	Constant Rotation Speed			Nonstationary Rotation Speed
	D1	D2	D3	D4
TDConvLSTM	**100%**	**100%**	**100%**	**97.56%**
TDConvLSTM Without BN	100%	99.5%	99.78%	93.11%
CNN-LSTM	98.67%	97%	98.33%	91.89%
CNN	96.83%	99.5%	98.17%	86.78%
LSTM	96.67%	99.83%	100%	80.94%
EMD-SVM	90.67%	89.67%	91.33%	75.67%

**Table 4:** Parameters of the proposed model used in tool wear monitoring experiments

No.	LAYER TYPE	Kernel	Stride	Channel	BN Axis	Activation
1	Local Convolution	(10,3)	(5,3)	4	4	ReLu
2	Local ConvLSTM	(2,2)	(1,1)	4	5	tanh
3	Holistic ConvLSTM	(4,4)	(1,1)	1	4	tanh
4	FC layer	10	-	1	−1	ReLu
5	Supervised learning layer	1	-	1	-	linear

**Table 5:** Mean absolute error (MAE) and root mean square error (RMSE) of models.

Model	MAE			RMSE		
	C4,C6/C1 ^1^	C1,C6/C4 ^2^	C1,C4/C6 ^3^	C4,C6/C1	C1,C6/C4	C1,C4/C6
TDConvLSTM	**6.99**	**6.96**	**7.50**	**8.33**	**8.39**	**10.22**
CNN-LSTM	11.18	9.39	11.34	13.77	11.85	14.33
CNN	15.32	14.34	17.36	18.50	18.80	21.85
LSTM	19.09	16.00	22.61	21.42	17.78	25.81

^1^ “C4,C6/C1” denote that C4 and C6 are the training datasets, C1 is the testing dataset. ^2^ “C1,C6/C4” denote that C1 and C6 are the training datasets, C4 is the testing dataset. ^3^ “C1,C4/C6” denote that C1 and C4 are the training datasets, C6 is the testing dataset.
